# Fisetin Alleviates Neointimal Hyperplasia via PPAR*γ*/PON2 Antioxidative Pathway in SHR Rat Artery Injury Model

**DOI:** 10.1155/2021/6625517

**Published:** 2021-04-21

**Authors:** Fang Pei, Hua Pei, Chunhua Su, Lin Du, Jifen Wang, Fusheng Xie, Qi Yin, Zhao Gao

**Affiliations:** ^1^Department of Cardiology, Bishan District People's Hospital in Chongqing Municipality, Chongqing 402760, China; ^2^Department of Cardiology, The Second Affiliated Hospital of Guilin Medical University, Guilin, Guangxi 541199, China; ^3^Clinical Lab, The Second Affiliated Hospital of Hainan Medical University, Haikou, Hainan 570331, China; ^4^Medical Laboratory, Xingyi People's Hospital, Xingyi, Guizhou 562400, China; ^5^Neurology Department, Xingyi People's Hospital, Xingyi, Guizhou 562400, China; ^6^Medical and Healthcare Center, Hainan General Hospital/Hainan Affiliated Hospital of Hainan Medical University, Haikou, Hainan 570311, China

## Abstract

The phenotypic transformation of proliferation and migration in vascular smooth muscle cells (VSMCs) from media to intima is the basic pathology of neointimal hyperplasia after angioplasty in hypertensive patients. Angiotensin II (AngII) stimulates oxidative stress in VSMC, inducing VSMC proliferation and migration, which is a critical factor in both developments of hypertension and angioplasty-induced arterial restenosis. Fisetin, a plant flavonoid polyphenol, has been reported to be antioxidative and potent senolytic. It is unknown whether fisetin would inhibit neointimal hyperplasia. Therefore, we investigated the role of fisetin in neointimal formation in vitro and in vivo. The rat thoracic aortic smooth muscle cells (A10 cells) stimulated by AngII were used as the in vitro neointimal hyperplasia model, where AngII significantly induced the proliferation and migration in A10 cells. We found that fisetin could dose-dependently inhibit the effect of AngII via inducing the expression of an antioxidant, paraoxonase-2 (PON2), whose overexpression could inhibit the proliferation and migration of A10 cells and downexpression by siRNA had the opposite effect. Furthermore, we found the mechanism of fisetin's inducing PON2 expression involved PPAR*γ*. Rosiglitazone, a PPAR*γ* agonist, could increase PON2 expression in A10 cells, while the PPAR*γ* inhibitor prevented the effect of fisetin on PON2. The in vivo neointimal hyperplasia model was established 2 weeks after the carotid artery balloon injury in SHR rats. Administration of fisetin (ip 3 mg/kg daily for 2 weeks) right after the injury significantly increased PON2 expression in the artery, inhibiting ROS production, and efficiently reduced carotid neointimal hyperplasia. These results indicate that fisetin increases the expression of antioxidant PON2 via activation of PPAR*γ*, reducing oxidative stress, inhibiting VSMC proliferation and migration, and alleviates neointimal hyperplasia after intimal injury. PON2 may be a potential therapeutic target to reduce arterial remodeling after angioplasty in hypertensive patients.

## 1. Introduction

Hypertensive patients undergoing angioplasty of percutaneous coronary intervention (PCI) are highly predisposed to vascular restenosis [1, 2]. Neointimal hyperplasia after vascular injury plays a critical role in the process of vascular restenosis, but the mechanism has not been fully elucidated yet [3]. It is commonly believed that abnormal proliferation and migration of medial vascular smooth muscle cells (VSMCs) are the pathological causes of neointimal formation after intima injury [4]. Angiotensin II (AngII), as the major effector of the renin-angiotensin-aldosterone system (RAAS), plays an important role in regulating blood pressure and arterial remodeling [5]. Via AT1a receptor, AngII increased oxidative stress, which is a critical regulator of VSMC proliferation and migration in hypertension and vascular remodeling [6–8]. Therefore, despite the existing anti-RAAS medicines, finding a novel downstream molecule exclusively targeting to inhibit neointimal formation and vascular remodeling may be a promising strategy for the treatment of vascular restenosis after angioplasty in hypertension.

Fisetin, a flavonoid phytonutrient present in almost all kinds of fruits and vegetables, has recently emerged as a powerful antioxidant for health promotion [9, 10] and exerts multiple protective effects in different oxidative stress-related conditions in human, such as degenerative diseases (e.g., vascular dementia), cancer, diabetes, and cardiovascular diseases [11–14].

Paraoxonase (PON) is an ancient enzyme showing native lactonase activity, catalyzes the hydrolysis of toxic organophosphates, and has been reported to be atheroprotective [15, 16]. Unlike its allozymes PON1 and PON3 are bound with HDL in circulatory vessels protecting LDL against oxidation, PON2 is ubiquitously located in subcellular structures, such as perinuclear region, endoplasmic reticulum (ER), and mitochondria, compartments that are crucial for balancing intracellular oxidative stress. PON2 is mainly involved in prevention of intracellular oxidative stress [17–19] and is an important endogenous defense mechanism against vascular oxidative stress in atherogenesis [16, 20–22]. In response to oxidative stress, PON2 dynamically translocates to the plasma membrane to fight against lipid peroxidation [23]. Besides the lactonase activity, other lactonase-independent mechanisms of PON2-mediated atheroprotection include inhibition of mitochondrial and ER stress-induced apoptotic pathways and mitochondrial-dependent superoxide generation [18, 24, 25]. In artery, PON2 was reported to be expressed at similar levels in three major human vascular cell types (i.e., endothelial cells, smooth muscle cells, and adventitial fibroblasts) where it may play a vascular-protective role [26]. We hypothesized that PON2 may serve as a potential target in the process of neointimal formation.

In the present study, we explored the protective effects of fisetin against VSMC proliferation and migration and its potential impact in preventing neointimal hyperplasia after angioplasty in hypertension. We also investigated its possible molecular mechanism on PON2-dependent antioxidative pathway.

## 2. Materials and Methods

### 2.1. Materials

Fisetin, AngII, rosiglitazone, and antibodies against ɑ smooth muscle actin (*α*-SMA) and osteopontin (OPN) were purchased from Sigma Co. (St. Louis, MO). Goat polyclonal antibody against PON2 was from Thermo Fisher Scientific (Waltham, MA). Proliferating cell nuclear antigen (PCNA) antibody was from Cell Signaling Technology (CST, USA). MAPK inhibitor PD98059, JNK inhibitor SP600125, and PPAR*γ* inhibitor GW9662 were from Selleck Chemicals (Houston, Texas, USA). SDS-polyacrylamide gels were from Pierce (Rockford, IL). Polyvinylidene fluoride (PVDF) and Western blotting-related apparatus were from Bio-Rad (Hercules, CA). All serum, cell media, and antibiotics were purchased from Thermo Fisher Scientific (Waltham, MA). All organic solvents were from Solarbio Life Sciences (Shanghai, China).

### 2.2. Cell Culture

A10 cell line derived from rat thoracic aortic arterial smooth muscle cells was obtained from ATCC (Hercules, CA). A10 cells were cultured in 10% FBS-DMEM containing 1% antibiotics and incubated in a CO_2_ incubator (5% CO_2_, 37°C). Cells were grown to 70 to 80% confluence (subconfluence) and then serum-deprived in 1% FBS for at least 6 hours. Quiescent A10 cells were treated with AngII in the presence or absence of indicated concentration of fisetin for 24 hours before further biochemical and cytological assays. Fisetin was dissolved in 1% dimethyl sulfoxide (DMSO). The cellular data were obtained from at least three independent experiments with three replicates performed in each trial.

### 2.3. Experimental Animals

SHR rats were purchased from Beijing Vital River Laboratory Animal Technology Co. The experimental protocol was approved by the Institutional Animal Care and Use Committee of Hainan Medical University (Haikou, China). All procedures were performed under appropriate anesthesia, and all efforts were made to minimize animal suffering.

### 2.4. Plasmid Transfection and siRNA Interference

For the transfection in A10 cells, PON2 plasmid was synthesized by Sangon Biotech Co., Ltd (Shanghai, China) and siRNAs by RIBOBIO (Guangzhou, China). When cells reached 80% confluence, plasid (1 *μ*g/mL) or siRNA (50 nM) was transfected by using Lipofectamine 2000 reagent (Thermo Fisher Scientific) in Opti-MEM™ (Thermo Fisher Scientific) according to the manufacturer's protocol. After 48 hours, the transfected cells were collected to determine the PON2 mRNA and protein levels or for cell proliferation and migration assays. The siRNA sequence with the best interfering effect was chosen and applied to our experiments.

### 2.5. Cell Proliferation Assay

Proliferation of A10 cells was determined by three different methods including 3-(4,5-dimethylthiazol-2-Yl)-2,5-diphenyltetrazolium bromide (MTT) (Beyotime Biotechnology, China), PCNA detection, and cell counting. A10 cells were seeded into 96-well culture plates (Corning, Lowell, MA) at a density of 2 × 10^3^ cells/well and were allowed to grow to subconfluence (70-80%) and then were serum starved for another 6 hours. Cells were divided into different groups corresponding to the indicated stimulus. After 24 hours, 10 *μ*l of MTT (5 mg/ml) was added to each well, and the incubation continued for an additional 4 hours at 37°C. Thereafter, 150 *μ*l of DMSO was added to each well, and the absorbance OD was read at 490 nm on a microplate reader (model 680, Bio-Rad). The level of DNA synthesis in A10 cells was detected by the measurement of PCNA expression via Western blotting and immunofluorescent staining [27]. The growth of A10 cells was also examined by cell counting. Cells were first made mitogenic quiescence by serum starvation in 1% FBS medium and then stimulated with indicated reagents at indicated times. The number of cells was counted in a hemocytometer after trypan blue staining (trypan blue uptake indicates cell death). Each count is an average of three repeats, and each data point is the average of three experiments.

### 2.6. Cell Migration Assay

Cell migration was examined by Transwell® and scratch-wound assays. The Transwell® migration assay was performed using 24-well tissue culture plates (BD Bioscience, Becton, NJ) with an 8 *μ*m pore polycarbonate membrane. The number of migratory cells was counted in 10 randomly chosen fields of duplicate chambers at a magnification of 200x for each sample.

For the scratch-wound migration assay, A10 cells were seeded in a 6-well plate at a density of 1 × 10^5^ cells/well, grown to subconfluence (70-80%), serum-starved for 6 hours, and then treated with indicated reagent. The cell monolayer was scratched with a small pipette tip along the ruler and then left to recover for the next 24 hours in freshly exchanged starvation medium (1% FBS DMEM). The images of the wounded area were captured immediately at 0 and 24 h after scratch to monitor VSMC migration into the wounded area under Olympus IX-70 inverted microscope (Olympus, Tokyo, Japan) using the Image J 1.44 software. The migration area was expressed as the fold difference in the number of migrated VSMCs compared with the corresponding control.

### 2.7. Cell ROS Detection

Reactive oxygen species (ROS) was detected by a fluorescent probe DCFH-DA (2,7-dichlorodihydrofluorescein diacetate). A10 cells were treated with AngII (10^−7^ mol/L) with or without fisetin (100 *μ*mol/L) for 24 hours. Culture medium was removed, and A10 cells were washed with cold PBS twice before the addition of 1 mL DCFH-DA (10 *μ*mol/L) (Beyotime Biotechnology). The fluorescence intensity was observed under an immunofluorescence microscope (Olympus IX71, Tokyo, Japan) and analyzed by Image J 1.44.

### 2.8. Protein Extraction and Western Blotting Analysis

A10 cells were washed once in PBS and then lysed in RIPA lysis buffer (Beyotime Biotechnology) containing a protease inhibitor mixture. Extraction of proteins, electrophoresis, transfer, immunodetection, and densitometric evaluation were performed as previously described [27]. Anti-PON2 antibody (1 : 500), anti-PCNA (1 : 500), anti-*α*-SMA (1 : 1000), and anti-OPN (1 : 1000) were used. The amount of protein transferred onto the membranes was normalized by immunoblotting with glyceraldehyde 3-phosphate dehydrogenase (GAPDH) (1 : 1000).

### 2.9. Quantitative RT-PCR

Total RNA was isolated from cells using TRIzol Reagent (Invitrogen, Carlsbad, CA, USA) following the manufacturer's instructions. Spectroscopy was then used to detect the concentration and purity of the RNA samples. QuantiTect Reverse Transcription Kit (Qiagen, Venlo, Netherlands) was used for reverse transcription of RNA to cDNA. Then, quantitative real-time PCR (RT-PCR) was performed with TB Green® Premix ExTaqII (Takara, Shiga, Japan). The mRNA expression level of PON2 gene was normalized by *β*-actin. The primer sequences for PON2 were 5′-TGGCTCTGAGTTTGCTAGGCA-3′ (forward) and 5′-TAAGTCGACTTCTGAGCGCCA-3′ (reverse). The primer sequences for *β*-actin were 5′-GTGGGTATGGGTCAGAAGGA-3′ (forward) and 5′-AGCGCGTAACCCTCATAGAT-3′ (reverse).

### 2.10. Carotid Balloon Injury Models in SHR Rats

SHR rats were anesthetized by intraperitoneal injection of 2.5% pentobarbital (50 mg/kg), and 100 U/kg heparin was injected into the tail vein. A median incision of 3-3.5 cm long in the neck was taken. The left common carotid artery, located next to trachea, was bluntly separated from the muscle with a vascular clamp. Then, the distal part of the artery including a small segment of the external carotid artery was completely isolated from the accompanying vagus and sympathetic nerves. A 2F Fogarty balloon catheter (Edwards Life Sciences, Irvine, CA, USA) was introduced into the common carotid artery through an arteriotomy in the external carotid artery and inflated to 1.0-1.5 atm. The balloon catheter was slowly moved back and forth 4 times to induce an intimal injury of about 10 mm long. After the skin suture, penicillin of 200,000 U was injected intramuscularly daily for 3 days to prevent infection [28]. SHR rats were injected intraperitoneally with either vehicle (1% DMSO; *n* = 6) or fisetin (3 mg/kg) daily for two consecutive weeks.

### 2.11. Evaluation of Neointimal Formation

To evaluate the morphometric grading of the neointimal formation, the SHR rats were euthanized by intraperitoneal injection of 2.5% pentobarbital (80 mg/kg), and 100 U/kg heparin was injected into the tail vein. The experimental common carotid arteries from SHR rats were immediately processed and embedded in 4% Paraformaldehyde and transected (4 *μ*m thick) in the middle segment of the injured or control common carotid arteries. Tissue sections were then stained with hematoxylin and eosin (HE). Fifteen sections from each carotid artery were observed under microscope. The intimal (I) and medial (M) areas were measured under 40x magnification using Image J 1.44 in a blind manner, and I/M ratio was calculated.

### 2.12. Determination of Malondialdehyde (MDA) and Total Cellular Antioxidant Capacity (T-AOC) in Carotid Aorta Artery

Homogenate of the whole common carotid artery was prepared with the sample preparation solution in ice-water bath and centrifuged at 4°C (12000 g, 5 min). Protein concentration was quantified according to the instruction of BCA Assay Kit (Beyotime Biotechnology). The level of MDA in the carotid artery was measured with a lipid peroxidation assay kit using the thiobarbituric acid method (Beyotime, Nanjing, China). Total antioxidant capacity (T-AOC) of the carotid artery was detected with a T-AOC assay kit using a rapid ABTS method (Beyotime, Nanjing, China).

### 2.13. Statistical Analysis

All data are expressed as mean ± SEM from at least three independent experiments and were analyzed using SPSS19.0 (IBM). One-way analysis of variance (ANOVA) was employed for comparisons among the experimental groups. Student's *t*-test was for comparison between only two groups. Statistically significant differences were defined as *P* < 0.05. All of the presented data were repeated at least three independent experiments.

## 3. Results

### 3.1. Fisetin Inhibited AngII-Induced Proliferation and Migration in Rat Aortic VSMCs

**Figure 1:**
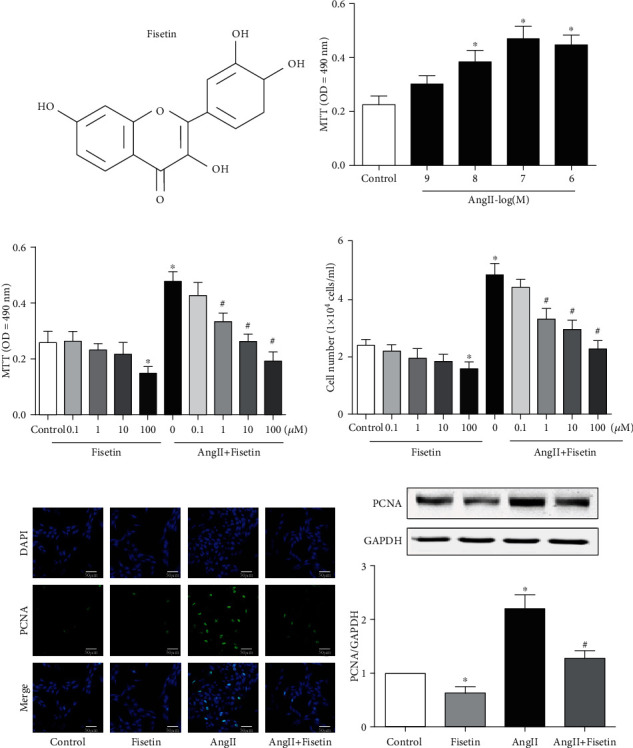
Fisetin inhibited AngII-induced proliferation in VSMCs. (a) Molecular structure of fisetin, a flavonoid compound found in plant tissues. (b) Effects of AngII on proliferation of VSMCs. A10 cells were treated with different concentrations (10^−9^~10^−6^ M) of AngII for 24 hours. Cell viability was determined by MTT assay. AngII with 10^−7^ M was chosen to induce a VSMC proliferation model in the upcoming experiments. (c–f) Effects of fisetin on AngII-induced proliferation in VSMCs. A10 cells were treated with different concentrations (0.1~100 *μ*M) of fisetin without or with AngII stimulation. Cell viability was determined by (c) MTT assay and cell (d) counting. Fisetin with 100 *μ*M was selected in (e) PCNA immunofluorescence staining and (b) PCNA immunoblotting. A representative image (scale bar = 50 *μ*m) and the relative intensity of PCNA immunoblotting were shown (^∗^*P* < 0.05 vs. control and ^#^*P* < 0.05 vs. AngII-only group).

The phenotypic transformation of proliferation and migration in VSMC plays an important role in hypertension and vascular remodeling. In order to observe the inhibitory effect of fisetin on VSMC proliferation, we first selected a proper concentration of AngII to consistently induce a model of VSMC proliferation, which was determined by MTT uptake. We can see AngII stimulated VSMC proliferation in a concentration-dependent manner (10^−9^~10^−6^ M) (Figure 1(b)). AngII with 10^−7^ M was chosen to induce a VSMC proliferation model in the upcoming experiments. Although fisetin, by itself, did not have an inhibitory effect on normal VSMC proliferation until a higher level of 100 *μ*M (among 0.1~100 *μ*M), it reduced AngII-mediated VSMC proliferation in a concentration-dependent manner from a lower level as 1 *μ*M (Figures 1(c) and 1(d)), as was also graphically demonstrated by immunofluorescence microscopy of PCNA expression and quantified by Western blotting of PCNA in VSMCs (Figures 1(e) and 1(f)).

**Figure 2:**
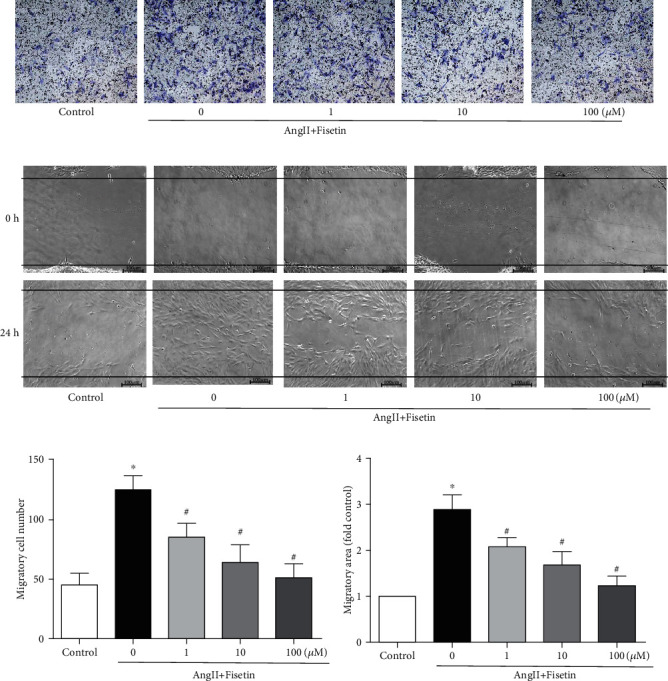
Fisetin inhibited AngII-induced migration in VSMCs. A10 cells were coincubated with incremental concentrations (0~100 *μ*M) of fisetin and AngII (10^−7^ M) in transwell assay and scratch-wound assay of VSMC migration. Representative images were shown, respectively, in (a) and (b) (scale bar = 100 *μ*m). Cell migration number in (c) transwell assay and migration distance in (d) scratch-wound assay were analyzed (^∗^*P* < 0.05 vs. control and ^#^*P* < 0.05 vs. AngII-only group).

The effect of fisetin on VSMC migration was also studied by transwell and scratch-wound migration tests. While AngII significantly increased VSMC migration, fisetin effectively inhibited AngII-mediated VSMC migration in a concentration-dependent manner (Figures 2(a) and 2(b)), which was quantitatively evaluated by migration cell counting and migration area calculation (Figures 2(c) and 2(d)).

### 3.2. Fisetin Inhibited AngII-Induced ROS Production, Cell-Proliferation, and Migration by Upregulating PON2 Expression in VSMCs

**Figure 3:**
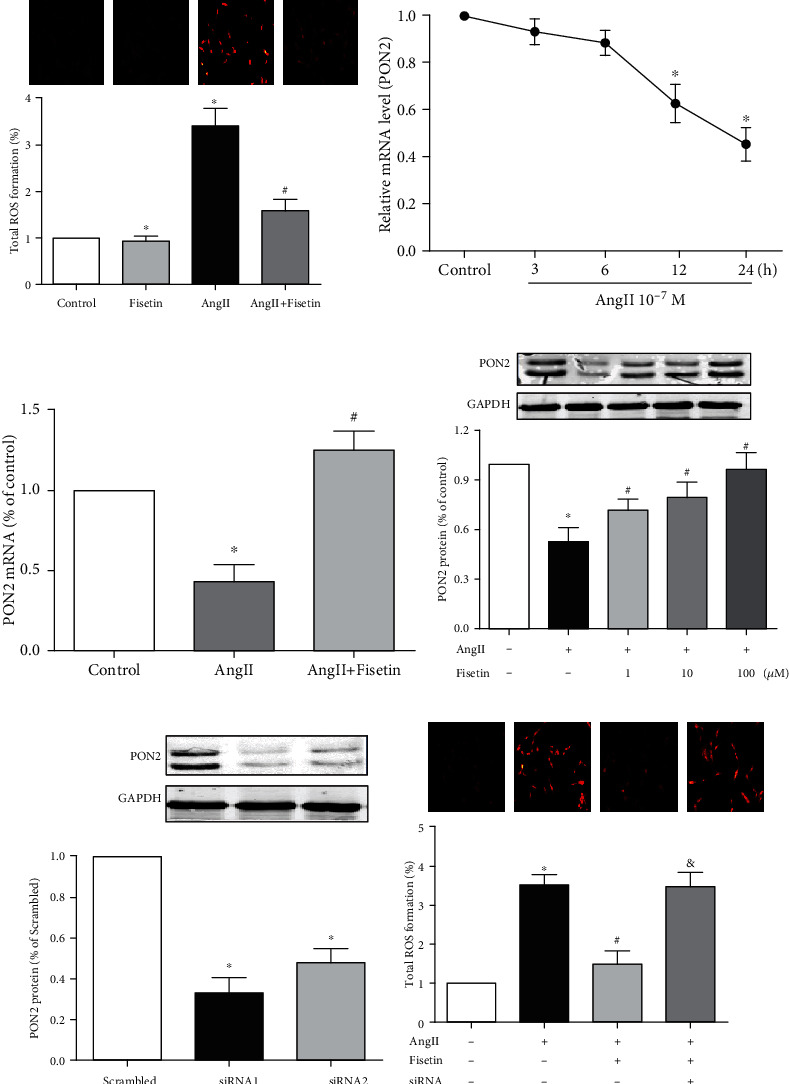
Fisetin inhibited AngII-induced ROS production in VSMCs through PON2. (a) Total ROS in cells was measured using the fluorescence probe DCFH-DA. Fluorescence intensity was analyzed by Image J. A10 cells were incubated with fisetin (100 *μ*M) or/and AngII (10^−7^ M) for 24 hours. (b) A10 cells were incubated with AngII (10^−7^ M) for incremental hours (3~24 h). PON2 expression in cells was determined by q-PCR. (c) A10 cells were treated with AngII (10^−7^ M)/and fisetin (100 *μ*M) for 24 hours before PON2 mRNA detection by q-PCR. (d) A10 cells were treated with incremental concentrations (0~100 *μ*M) of fisetin and AngII stimulation. Protein expression of PON2 was determined by immunoblotting. (e) A10 cells were transfected with different PON2 siRNAs for 48 hours. PON2 protein expression was determined by Western blotting. siRNA1 was selected for subsequent PON2 interference test. (f) PON2 siRNA-transfected A10 cells were coincubated with AngII and fisetin for 24 hours. Total ROS production was compared with normal PON2 expression cells. Representative images were shown (^∗^*P* < 0.05 vs. control, ^#^*P* < 0.05 vs. AngII only, and ^&^*P* < 0.05 vs. no siRNA).

Extensive researches have shown that fisetin plays a protective role in various organs by inhibiting oxidative stress. We also confirmed that fisetin can reduce the total ROS production in AngII-stimulated VSMCs (Figure 3(a)), but little is known whether it could regulate PON2, a ROS-scavenging molecule. Therefore, we examined the changes of PON2 expression first. As shown in Figures 3(b) and 3(c), respectively, within 24 hours AngII time-dependently reduced the mRNA expression of PON2 in VSCMs, but the downregulation of PON2 gene transcription was reversed in the presence of fisetin. Western blotting also confirmed that fisetin concentration-dependently reversed the expression of PON2 at protein level (Figure 3(d)).

To further clarify whether PON2 plays a role in fisetin's downregulation of ROS generation in VSCMs, PON2 siRNA was employed to interfere with the expression of PON2. The interference effects of candidate siRNAs are shown in Figure 3(e). siRNA with the best interference effect was selected for subsequent PON2 interference test. Our results showed that the silence of PON2 expression eliminated the inhibitory effect of fisetin on ROS generation (Figure 3(f)). These results indicate that PON2 is a downstream signal of fisetin and plays an important role in fisetin's inhibitory effect on ROS generation in VSCMs.

**Figure 4:**
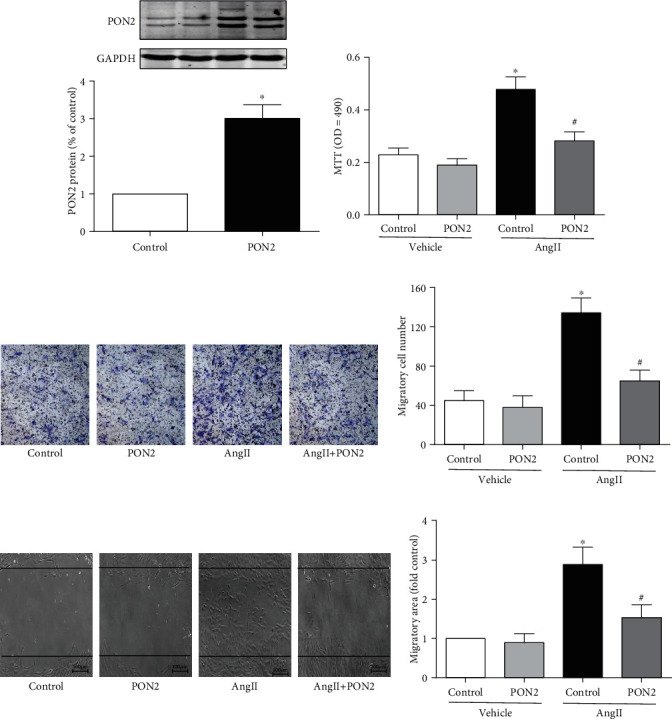
Overexpression of PON2 inhibited AngII-induced proliferation and migration in VSMCs. (a) A10 cells were transfected with plasmids carrying PON2 overexpression genes. PON2 protein expression was determined by Western blotting. (b–d) PON2 overexpression A10 cells were treated with AngII (10^−7^ M) for 24 hours. Cell proliferation was determined by (b) MTT assay. Cell migration was determined by (c) transwell assay and (d) scratch-wound test. Representative images were shown, respectively. Cell migration number and migration distance were also analyzed (^∗^*P* < 0.05 vs. control and ^#^*P* < 0.05 vs. AngII only).

PON2 reduces oxidative stress in vascular cells and decreases ER stress-induced caspase activation, thereby protecting vascular function and preventing atherosclerosis [21]. However, whether PON2 could inhibit the proliferation and migration of VSMCs has not been reported. So, we constructed a cell line overexpressing PON2 by plasmid transfection carrying PON2 gene, as shown in Figure 4(a). We found that PON2 overexpression reversed AngII-induced VSMC proliferation tested by MTT assay (Figure 4(b)) and reduced VSMC migration by transwell and scratch-wound migration tests (Figures 4(c) and 4(d)).

### 3.3. Fisetin Upregulated PON2 Expression through PPAR*γ* Signaling Pathway

**Figure 5:**
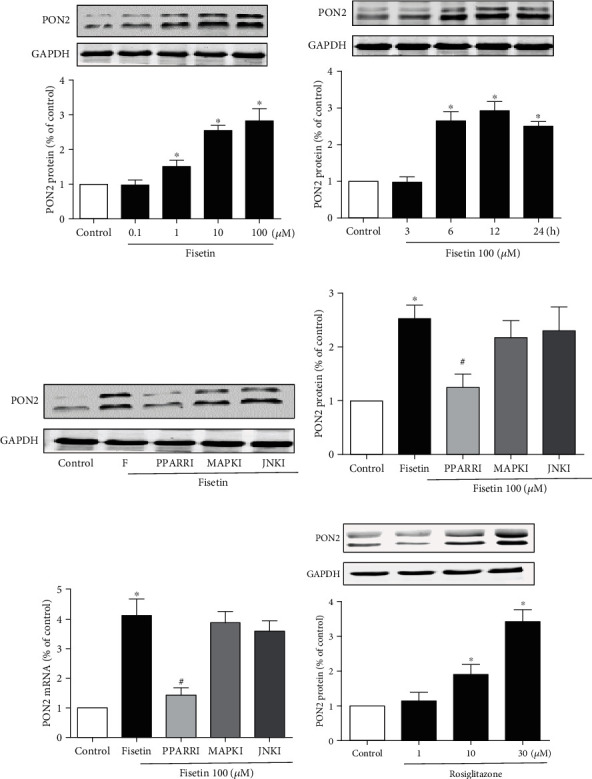
Fisetin regulates PON2 expression through PPAR*γ* pathway in VSMCs. (a, b) Effects of fisetin with incremental doses or incremental incubation times on PON2 protein expression in the absence of AngII. Protein level was determined by Western blotting after A10 cells were incubated with incremental concentrations (0.1~100 *μ*M) of fisetin alone for 24 h or with a fixed concentration of fisetin (100 *μ*M) for incremental times (3~24 h). (c, d) Role of PPAR*γ* in fisetin's upregulation of PON2 expression in VSMCs. A10 cells were treated with 100 *μ*M of fisetin for 24 hours in the presence of either PPAR*γ* inhibitor GW9662 (10 mM) or MAPK inhibitor PD98059 (10 mM) or JNK inhibitor SP600125 (1 mM). The expression of PON2 was detected by (c) Western blotting and (d) q-PCR. (e) Effect of PPAR*γ* agonist rosiglitazone on protein expression of PON2. A10 cells were treated with incremental concentrations of rosiglitazone (10~30 *μ*M) for 24 hours before PON2 protein immunoblotting (^∗^*P* < 0.05 vs. control and ^#^*P* < 0.05 vs. fisetin only).

To further explore the mechanism of the upregulation effect of fisetin on PON2, firstly, we should determine an experimental condition with proper concentration of fisetin and proper incubation time and found that fisetin upregulated the protein expression of PON2 in both concentration-dependent and time-dependent manners in the absence of AngII (Figures 5(a) and 5(b)). Fisetin with concentration of 100 *μ*M incubated with VSMCs for 24 hours was selected for subsequent tests. To determine through which signaling pathway fisetin may regulate the expression of PON2, based on literature reviewing, we screened several classic downstream signaling inhibitors, including PPAR*γ*, MAPK, and JNK inhibitors. Results revealed that PPAR*γ* inhibitor (GW9662) effectively blocked the upregulation effect of fisetin on PON2 protein and mRNA expression (Figures 5(c) and 5(d)). In addition, the PPAR*γ* agonist rosiglitazone has a similar effect as fisetin in increasing the protein expression of PON2 (Figure 5(e)).

**Figure 6:**
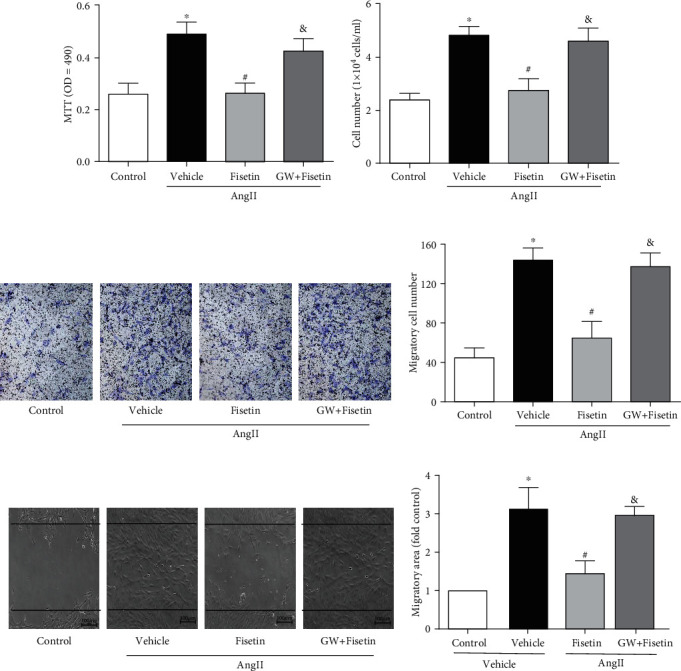
PPAR*γ* inhibitor eliminated the inhibitory effect of fisetin on AngII-induced VSMC proliferation and migration. A10 cells in the presence of AngII were incubated with fisetin only or fisetin plus the PPAR*γ* inhibitor GW9662 (GW) for 24 hours. Cell proliferation ability was evaluated by (a) MTT assay and (b) cell counting. The migration ability of VSMCs was determined by (c) transwell assay and (d) scratch-wound tests. Representative images were shown. Cell migration number and migration distance were also analyzed, respectively (^∗^*P* < 0.05 vs. control, ^#^*P* < 0.05 vs. AngII only, and ^&^*P* < 0.05 vs. fisetin with no GW).

To clarify the role of PPAR*γ* in the effect of fisetin on VSMC proliferation and migration, we employed the PPAG*γ* inhibitor GW9662 to coincubate with fisetin. Results showed that PPAG*γ* inhibitor almost reversed the inhibitory effect of fisetin on AngII-induced VSMC proliferation verified by MTT assay and cell counting (Figures 6(a) and 6(b)) and VSMC migration by transwell and scratch-wound migration tests (Figures 6(c) and 6(d)). Above evidence showed that fisetin inhibited the proliferation and migration of VSMCs by upregulating the expression of PON2 through PPAR*γ* signaling pathway.

### 3.4. Fisetin Inhibited Neointimal Hyperplasia In Vivo

**Figure 7:**
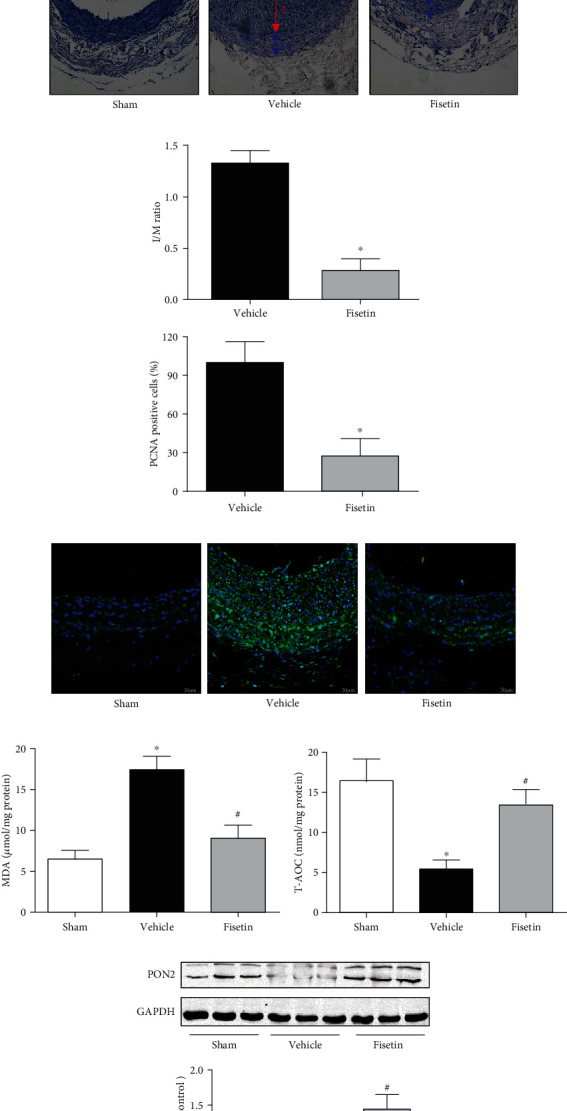
Fisetin inhibited neointimal hyperplasia after balloon injury of carotid artery in SHR rats. Neointimal hyperplasia of carotid artery was induced after balloon injury in SHR rats. Fisetin (3 mg/kg) or vehicle was intraperitoneally injected daily for two weeks. Then, carotid artery was harvested for neointima examination. (a) Representative images of hematoxylin-eosin (HE) cross-sections in different groups were shown at 10x and 40x magnifications. (b) The ratio of intima (I) to media (M) was compared between vehicle and fisetin groups. (c) Representative immunofluorescence images of PCNA (green) and DAPI (blue) (scale bar = 20 *μ*m). The percentage of PCNA-positive cells counted under 40x magnification was compared (^∗^*P* < 0.05 vs. vehicle, *n* = 5/group). The level of (d) MDA and (e) T-AOC (total cellular antioxidant capacity) activity in carotid artery were measured. (f) The effect of fisetin on PON2 protein expression in carotid artery determined by Western blotting (^∗^*P* < 0.05 vs. sham and ^#^*P* < 0.05 vs. vehicle, *n* = 5/group).

In order to further verify the preventive effect of fisetin on neointimal hyperplasia in vivo, we established a balloon-injured carotid artery model in SHR rats to simulate neointimal formation after PCI in hypertensive patients. The arterial HE staining showed that fisetin significantly reduced the thickness of intima after injury, as the ratio of intima to media was greatly reduced (Figures 7(a) and 7(b)). PCNA immunofluorescence staining of arterial wall demonstrated that the proliferation ability of vascular cells triggered by balloon injury was markedly increased and effectively suppressed by administration of fisetin, as the number of PCNA-positive cells was significantly reduced (Figure 7(c)). At the same time, the contractile phenotype of smooth muscle was evidently degenerated after injury, since the biomarker *α*-SMA was significantly reduced and OPN increased by Western blotting, while administration of fisetin prevented these changes of *α*-SMA and PON, indicating an effect of preserved contractile phenotype by fisetin (Figure S1 in the Supplementary Material). We also tested the oxidative level in the artery and found that fisetin counteracted the oxidative stress in the injured arteries, as manifested by reduced MDA level and restored T-AOC activity (Figures 7(d) and 7(e)). The reverse and upregulation effect of fisetin on PON2 expression in injured arteries was confirmed by Western blotting as in vitro study (Figure 7(f)).

## 4. Discussion

Nowadays, the morbidity of cardiovascular diseases still continues increasing, and coronary artery disease is a major concern [29]. While PCI has been widely performed to treat coronary atherosclerotic heart disease, coronary restenosis may occur after angioplasty, which would definitely compromise the initial success of PCI, affecting the long-term prognosis and bringing new cardiovascular risks to postoperative patients [1, 2]. Although prophylactic and therapeutic methods, such as new generation of drug-eluting stents and bioresorbable scaffold stents, have been developed to reduce restenosis after angioplasty, the problem is far from being resolved [30]. Due to its complexity, the underlying mechanism of restenosis still has not been fully elucidated after decades of research. At present, it is widely believed that abnormal neointimal hyperplasia during the repair of injured vessels is the basic pathophysiological change of restenosis after PCI [3, 31]. The enhanced proliferation and migration ability of VSMCs, transferred from the media to intima, is an important cause of neointimal formation after vascular injury [3]. Hypertension also plays an important role in coronary restenosis and has been proven as an independent predictor of restenosis in coronary heart disease patients after stent implantation [1]. Antihypertensive drugs such as valsartan are believed to inhibit neovascularization [32]. In this study, we established the animal model of neointimal hyperplasia in SHR rats.

Epidemiological studies have already confirmed that fruits and vegetables can reduce the risk of cardiovascular diseases, wherein the antioxidant property of phenolic compounds plays an important role [33, 34]. Phenolic compounds have constantly been shown to reduce blood pressure and inhibit vascular restenosis caused by hypertension and intimal injury [35]. Fisetin, as a flavonoid compound, has also been extensively studied because of its antioxidant property, exerting various biological effects such as anti-inflammatory, antitumor, and cardiovascular protection [9–14]. It is remarkable that, among the 10 flavonoids tested, including resveratrol, epigallocatechin gallate, quercetin and curcumin, fisetin was the most potent senolytic, which could reduce senescence markers in multiple tissues [36]. Fisetin could relax smooth muscle by regulating calcium-dependent signals, but it is unclear whether it can regulate the proliferation and migration of smooth muscle cells or not. Our study found that the proliferation and migration of VSMCs induced by AngII could be suppressed in the presence of fisetin in time- and dose-dependent manners in vitro.

At one hand, studies have demonstrated the various biological functions of fisetin are mainly achieved through its antioxidant capacity [9]. At the other hand, we know ROS or oxidative stress is largely involved in the pathophysiological processes of atherosclerosis and restenosis, and reducing ROS can inhibit the proliferation and migration of VSMCs [37, 38]. We wondered whether fisetin could reduce ROS production in VSMCs and measured the total ROS in VSMCs to find that fisetin significantly reduced ROS production caused by AngII stimulation. We further searched for the possible downstream effector molecules by which fisetin may regulate oxidative stress. Paraoxonase-2 (PON2), an intracellular antioxidant enzyme, has been shown to be regulated by several polyphenolic compounds [39]. Previous studies also have shown that fisetin can prevent LDL from being oxidized and block ox-LDL from being taken up by macrophages [40], and this effect is highly similar to the function of the paraoxonase family. So, we speculated that the antioxidant effect of fisetin may achieved by regulating the expression of PON2.

Paraoxonase (PON) is a member of the lactonase family and contains three allozymes: PON1, PON2, and PON3. PON1 and PON3 are mainly expressed in liver and circulating in vascular vessels, and PON2 is widely distributed throughout the body in various cells [41–43]. PON2 can reduce the production of oxidized LDL and peroxides in cells, thereby reducing oxidative stress. Our results confirmed that AngII suppressed the expression of PON2, and fisetin can reverse this effect. By using siRNA, we constructed a PON2 downregulated cell line, further verified that silence of PON2 gene can block the inhibitory effect of fisetin on ROS generation induced by AngII in VSMCs. All above taken together indicate that PON2 plays an important role in the antioxidant effect of fisetin.

By literature reviewing, we found fisetin can activate multiple signaling pathways including JNK, PPAR*γ*, and p38MAPK [40, 44, 45]. To further elucidate in which pathway fisetin may regulate the expression of PON2, we screened these classic signaling inhibitors and found that GW9662, an inhibitor of PPAR*γ* signaling pathway, effectively blocked the upregulation effect of fisetin on PON2 expression. In addition, rosiglitazone, a PPAR*γ* agonist, can upregulate the expression of PON2 like fisetin. It suggested that PPAR*γ* is a downstream signaling pathway by which fisetin regulates the expression of PON2.

PON2 gene polymorphism is associated with the incidence of cardiovascular diseases [46, 47]. The vascular protective effect of PON2 has been well acknowledged. The protective mechanism involves functions such as antioxidation, regulation of inflammatory factors, and improvement of mitochondrial and endoplasmic reticulum stress [14, 17, 21]. However, whether PON2 could affect the proliferation and migration of VSMCs has not been reported yet. By constructing PON2 siRNA silence and plasmid overexpression VSMC cell lines and then incubating with AngII, we confirmed the inhibitory effect of PON2 on VSMC proliferation and migration. In vivo experiments also proved the results of cell tests. Fisetin significantly inhibited neointimal hyperplasia caused by balloon injury in carotid arteries of SHR rats, prevented phenotypic transformation of arterial smooth muscle cells with preserved contractile phenotype, and upregulated the arterial expression of PON2.

In this study, we used AngII to induce VSMC proliferation and migration and constructed a balloon injury-induced neointimal hyperplasia model in carotid artery in SHR rats. The above results proved that fisetin can produce a protective effect in vascular remodeling in vivo and in vitro and indicated that fisetin upregulates the expression of PON2 by activating PPAR*γ* signaling pathway, reduces oxidative stress in VSMCs and vessel lesions, represses VSMC proliferation and migration, and significantly prevented neointimal hyperplasia caused by AngII and balloon injury. However, there still exist some problems that need further research and explanation, for example, whether the regulation of VSMCs by fisetin is related to its lactase activity. Can fisetin play a protective role independent of its antioxidant capacity? In conclusion, our results defined PON2 as a downstream target of fisetin and confirmed that PON2 plays a regulatory role in the proliferation and migration of VSMCs. To our knowledge, this result has not been reported. Our research provides new insights into the protection of cardiovascular systems by natural plant compounds.

## 5. Conclusions

This study revealed a crucial role of fisetin in balloon injury-induced neointimal hyperplasia in SHR rats. We are the first to show that fisetin can attenuate neointimal formation through its antiproliferation and antimigration effects on VSMCs via PPAR*γ*/PON2 pathway. Fisetin/PON2 could be a potential therapeutic target to prevent vascular restenosis after angioplasty in hypertensive patients.

## Data Availability

The research article data used to support the findings of this study are included within the article.

## Supplementary Materials

Supplementary Materials Figure S1: fisetin prevented phenotypic transformation and preserved the contractile phenotype in balloon-injured artery. Neointimal hyperplasia of carotid artery was induced after balloon injury in SHR rats. Fisetin (3 mg/kg) or vehicle was intraperitoneally injected daily for two weeks. Then, carotid artery was harvested. The effects of fisetin on biomarkers (a) *α*-SMA and (b) OPN of contractile phenotype in carotid artery were determined by Western blotting (^∗^*P* < 0.05 vs. sham and ^#^*P* < 0.05 vs. vehicle, *n* = 5/group).
