# Supplementary material for: Fisetin Alleviates Neointimal Hyperplasia via PPARγ/PON2 Antioxidative Pathway in SHR Rat Artery Injury Model
Source: Oxid Med Cell Longev. 2021 Apr 21;2021:6625517. doi: 10.1155/2021/6625517 (PMC8084648; doi:10.1155/2021/6625517)
Supplement: Supplementary Materials — Figure S1: fisetin prevented phenotypic transformation and preserved the contractile phenotype in balloon-injured artery. Neointimal hyperplasia of carotid artery was induced after balloon injury in SHR rats. Fisetin (3 mg/kg) or vehicle was intraperitoneally injected daily for two weeks. Then, carotid artery was harvested. The effects of fisetin on biomarkers (a) α-SMA and (b) OPN of contractile phenotype in carotid artery were determined by Western blotting (∗P < 0.05 vs. sham and #P < 0.05 vs. vehicle, n = 5/group). [file 6625517.f1.pdf]

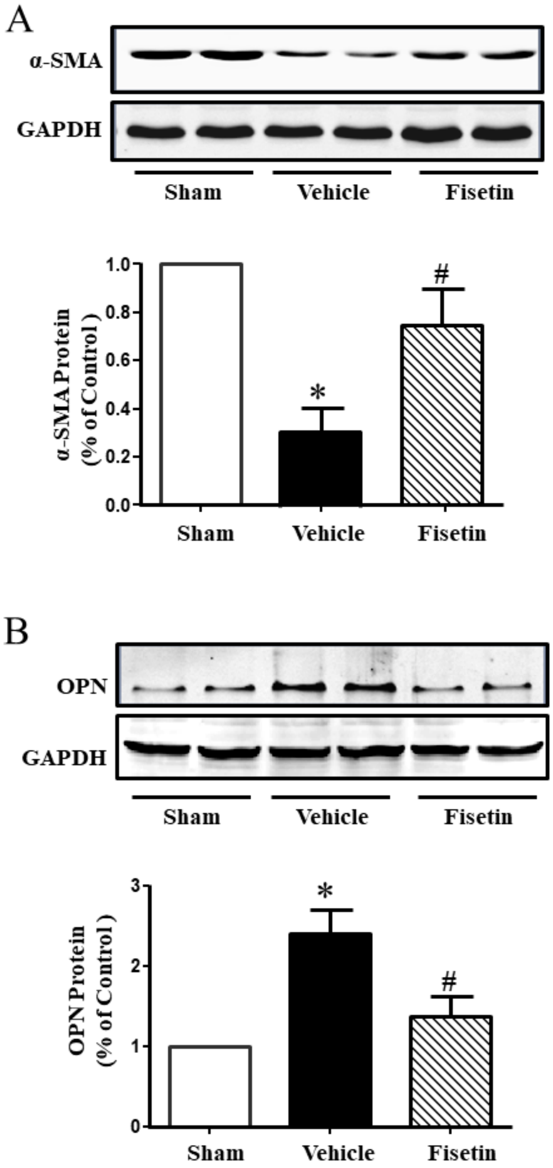

Figure S1. Fisetin prevented phenotypic transformation and preserved the contractile phenotype in balloon injured artery.

Neointimal hyperplasia of carotid artery was induced after balloon injury in SHR rats. Fisetin (3 mg/kg) or vehicle was intraperitoneally injected daily for two weeks. Then, carotid artery was harvested. The effects of fisetin on biomarkers α-SMA (a) and OPN (b) of contractile phenotype in carotid artery were determined by western blotting (*P<0.05 vs. sham, #P<0.05 vs. vehicle, n=5/group).
